# Social scientists’ testimony before Congress in the United States between 1946-2016, trends from a new dataset

**DOI:** 10.1371/journal.pone.0230104

**Published:** 2020-03-25

**Authors:** Thomas V. Maher, Charles Seguin, Yongjun Zhang, Andrew P. Davis

**Affiliations:** 1 Department of Sociology, Purdue University, West Lafayette, Indiana, United States of America; 2 Department of Sociology, Penn State University, State College, Pennsylvania, United States of America; 3 School of Sociology, University of Arizona, Tucson, Arizona, United States of America; 4 Department of Sociology, North Carolina State University, Raleigh, North Carolina State, United States of America; Wuhan University, CHINA

## Abstract

Congressional hearings are a venue in which social scientists present their views and analyses before lawmakers in the United States, however quantitative data on their representation has been lacking. We present new, publicly available, data on the rates at which anthropologists, economists, political scientists, psychologists, and sociologists appeared before United States congressional hearings from 1946 through 2016. We show that social scientists were present at some 10,347 hearings and testified 15,506 times. Economists testify before the US Congress far more often than other social scientists, and constitute a larger proportion of the social scientists testifying in industry and government positions. We find that social scientists’ testimony is increasingly on behalf of think tanks; political scientists, in particular, have gained much more representation through think tanks. Sociology, and psychology’s representation before Congress has declined considerably beginning in the 1980s. Anthropologists were the least represented. These findings show that academics are representing a more diverse set of organizations, but economists continue to be far more represented than other disciplines before the US Congress.

## Introduction

The United States Congress (henceforth simply “Congress”) plays a vital role in the legislative process in the United States. The congressional hearing is a crucial part of Congresses’ process of drafting and evaluating legislation where stakeholders and experts are invited to testify before lawmakers. In an ideal world, Congress would invite social scientists to testify on relevant social matters, but we know relatively little about the extent to which social scientists appear at congressional hearings. What we do know suggests that many prominent channels of government influence and policy making have been captured by economists and economic models [1–6], and the most comprehensive comparative data on the subject finds that economists are more likely than other social science disciplines to be *mentioned* in the congressional record after 1989 [7]. In this article, we describe new—nearly comprehensive and publicly available—data describing when anthropologists, economists, political scientists, psychologists, and sociologists testified before the United States’ Congress from 1946 through 2016 (the data and a codebook are publicly available [8]).

We also describe data showing that the organizational sources of expertise have diversified over time [9]. The boundaries between science and politics are often fuzzy [10], and this is evident in the sources of social science testimony. Social scientists testify in university-affiliated roles, as government officials, business corporations, labor unions, other interest groups, or for think tanks. Like scientists more broadly [11], social scientists representing think tanks began receiving invitations in the 1960s, and they have become an increasingly central source of social science representation in the 21^st^ century. Economists testify much more often regardless of their organizational affiliation, giving more than twice as much testimony as any of the other disciplines, but political scientists also increasingly appear before Congress as representatives of think tanks.

Below, we briefly review work on the causes and impacts of congressional testimony, describe our data collection, and describe trends in Congressional representation in some detail. Our goal is not to explain social scientists’ representation before Congress, nor is our goal to explain what influence these hearings have on government processes, legislative or otherwise. Our goal is to provide a broad empirical overview, and introduce new data that can be of use for future explanatory work [3,12]. We conclude with some potential directions for research.

## The role of congressional hearings in the United States’ legislative process

The United States Congress is composed of two chambers of elected officials: the House and the Senate. Both the House and Senate draft and vote to approve new legislation. For proposed legislation to become passed into law, both the House and Senate must approve it by a majority vote. Even in cases where the US president vetoes such legislation, the veto can be overridden if both the House and Senate vote by a two-thirds majority in favor of the legislation. As such, Congress has tremendous influence over the legislative process. There is a long history of politicians drawing on scientists to gather information on new policies, or to provide support for existing policy proposals [3,10,13,14]. Nonetheless, citizens (and politicians) in the United States have varying levels of trust in scientific expertise [15]. Understanding what types of information the US Congress draws on to make decisions, and how information sources have changed, offers insights into the role of scientific expertise for government officials in a politically and cultural central country in the global system [16,17].

Congress has several tools for gathering information and analyzing policy at its disposal. In addition to institutions like the General Accountability Office, Congressional Budget Office, and Office of Technology Assessment, Congress also holds five non-mutually exclusive types of hearings: legislative hearings on specific bills and legislation (although this does not guarantee that the issue will be voted on); oversight hearings to review, monitor, and supervise public policy; investigative hearings to assess allegations of wrongdoing; confirmation hearings for nominated officials; and field hearings that are held off-site. Majority and minority members on House and Senate congressional committees can call or subpoena witnesses for hearings, and typically call hundreds of hearings each year. Between 1946 and 2016, roughly 34% of hearings were called to debate specific bills while 66% of hearings were called to gather information more generally. In sum, Congress called 879,960 witnesses to testify at these hearings, and these witnesses represent an array of groups and interests, including local and state politicians, police, industry and business interests, religious groups, social movement organizations, and the social sciences.

While scholars recognize that laws generally follow hearings [18], there is disagreement over the exact role that hearings play in the legislative process, and there is no clear straight line between congressional testimony and influence on legislation. Although hearings can be largely symbolic, many argue that members of Congress can use hearings to signal the importance of an issue or strategically invite witnesses to draw attention to problems with pieces of legislation [19,20]. Baumgartner and Jones contend that Congress uses hearings in two ways: to draw on witnesses’ technical expertise for the bill-writing process, and to make sense of a diversity of information (i.e. “entropic information”) when setting priorities [18]. We emphasize that our data cannot, on their own, speak to how social scientific testimony is being used, or what influence it might have, although our hope is that these data will be useful for future research on these questions.

## Data collection

We gathered data on congressional testimony through the publicly available list of invited speakers present in the congressional record. Specifically, we used ProQuest’s Congressional Hearing database, made available through the University of Arizona. We web-scraped each listed hearing from 1946–2016 for detailed metadata. The database contains full lists of the committees and sub-committees which convened the hearing, the date of the hearing, as well as information about each of the witnesses that testified before each congressional hearing, including their names, organizational affiliation, and titles. Our search complies with ProQuest’s Terms and Conditions because the search is for research and analysis purposes, uses only reasonable portions of the data, (here, data on social scientists who testify), and the underlying dataset only shares data for a portion of the material without replacing future users’ need to work through ProQuest (or other points of access to the government record) to access the full scope of the data. Further, the material we collect (hearing dates, topics, and witness lists) is publicly available through several other sources; we do not collect or use ProQuest’s proprietary transcripts of the testimony. Moreover, since these data points are facts, they are not subject to US copyright. Our web-scraping procedure resulted in a dataset containing every congressional hearing and witness from 1946 to 2016. These included a total of 91,809 hearings and 879,960 different testimonies.

We then systematically searched through the witness records to identify social scientists from five core disciplines over time: anthropology, economics, political science, psychology, and sociology. Although these disciplines are central social science departments in most universities, they do not exhaust the intellectual or organizational spaces occupied by social scientists. These disciplines were well-established by 1946, but the years since have witnessed the rise of ethnic studies [21], women’s studies [22], American studies [23], and the increasing embrace of the social sciences by business schools [24], among other organizational changes. These new schools, departments, and centers initially started by employing scholars from existing disciplines, but have begun training and hiring their own PhDs. Social science disciplines tend, however, to only hire scholars with PhDs from their own discipline [23]. Interdisciplinarity has also risen as an intellectual style over this period [25–27]. Moreover, disciplines themselves are dynamic [23], so scholars from the same discipline, but at different time periods, will also be quite different. We focus on the testimony of scholars with PhDs or Master’s degrees granted by one of these five disciplines. By focusing on the disciplinary source of their degrees, rather than current affiliations, we capture many scholars working in interdisciplinary departments or intellectual traditions, although of course this remains a moving target.

To identify witnesses, we first identified basic search terms such as “sociologist,” “professor of economics,” “department of psychology,” etc. and used these to search the witnesses’ affiliation (see the codebook for a full list of basic search terms). We developed these through an iterative process, discovering which terms identified witnesses from our three disciplines, and which yielded false positives. For illustration, we found that the term “sociolog*” found witnesses containing “sociologist” or “professor of sociology” in their title, but rarely witnesses who were not sociologists. For example, James Coleman was described as affiliated with the “sociology dept, Univ of Chicago” when he testified in 1985 before the Task Force on Science Policy regarding the applicability of social science research to questions of policy, and would be classified as a sociologist through our keyword search. We then manually reviewed all cases where the witness was from a combined department (e.g., Department of Sociology and Anthropology) to determine the witnesses’ core discipline using the protocol described below.

Second, we searched witness records for the names of prominent members of our five disciplines which may have been missed when witnesses’ affiliations did not include any disciplinary identifiers. James Coleman, again, for example, is listed as representing the National Opinion Research Center when he testified before the House Education and Labor Committee about reviewing Office of Educational Research and Improvement educational R&D programs in 1988. Searching for prominent individuals by name ensures that we identify as many social scientists as possible, regardless of their witness description. To identify prominent social scientists, we collected a list of potential witnesses that had any public Wikipedia webpage. In sum, 656 American sociologists, 1,923 American psychologists, 969 American political scientists, 1,853 American economists, and 948 American anthropologists were included. We checked this list against Google Scholar’s profile search with the labels: “sociology,” “psychology,” “political science,” “economics,” and “anthropology” to correct any wrongly categorized or missing people. After our search was completed, supervised undergraduate coders reviewed the congressional witnesses identified based on each disciplinary list and separated matches and false positives; fifty-five percent (3847) of the initial 6965 matches were false positives. The proportion is similar if we focus on individual witnesses as potential false positives. Roughly 50% of the 1260 individuals flagged were false positives. Forty-three percent (544) were positives. The remaining seven percent were cases where multiple individuals had the same, or similar, names. The number of false positives during this stage is due in part to our decision to include everyone with a similar first and last name. For example, we collected everyone named “William Wilson” when searching for whether William Julius Wilson testified (he did, along with 11 other people named “William Wilson”).

Third, scientific expertise has democratized over the past half century [28]. Indeed, social science expertise increasingly comes from non-academic sources [9,29], and so our next step was to systematically review lists of individuals associated with social science adjacent government and non-government institutions to identify relevant witnesses. We began by reviewing any witness in the congressional record that was associated with a think tank. Think tanks have become a prominent point of contact between politics and the academy, and Congress and political officials are a primary audience for liberal and conservative think tanks to express their ideas [29]. Indeed, it is their ability to work across multiple different fields—academia, politics, business, and the media—that gives them cultural influence [30,31] and embodies the politicization of science and expertise [28,32]. We identified every individual who testified on behalf of one of 205 prominent think tank organizations where social scientists might work and be classified as representatives before Congress. We gathered our list of think tanks from Wikipedia, and the authors manually reviewed the list to ensure that it included all of the major organizations in other lists [29,33]. While this may include social scientists who testified on behalf of “questionable” think tanks, we are confident that no major think tank representatives were omitted from the analysis.

We trained undergraduate coders to research each individual whose discipline was not listed in the congressional record or who was not on a disciplinary list in order to determine if they had earned an advanced degree in one of our five social science disciplines (i.e. master’s degree or Ph.D). This primarily applied to think tanks and government representatives, but included some academics (e.g., individuals listed affiliated with public policy positions) as well. We coded affiliated fields as matches for our five disciplines of interest. Archaeology was coded as anthropology; finance, business and business administration PhDs (not MBAs) were coded as economics; therapy was coded as psychology; and demography and criminology were coded as sociology. We coded public policy, government, international relations, and national security as political science. Public policy turned up several false positives in our initial search, and so we manually coded all individuals with “public policy” in their witness descriptions in order to distinguish individuals from schools of public policy (e.g., Judith Feder, a professor of public policy at Georgetown) from those who were from the public policy divisions of other organizations (e.g., Nancy Chapman, the public policy director for the Society for Nutrition Education).

We acknowledge that the boundaries around academic fields are fuzzy and often overlapping, especially as universities offer more interdisciplinary training [34,35]. We attempted to identify interdisciplinary scholars of Science and Technology Studies using the method described above, but only found five experts who list STS as their affiliation, and one, Elting Morison, from a list of prominent STS scholars on Wikipedia. Similarly, we found only 11 results for “Development Studies” affiliated scholars indicating that Congress is not inviting these scholars to testify in significant numbers compared to the traditional disciplines. Of course, like other fields, their impact may be stronger in other areas of the policymaking field. We include full details about disciplinary decision making in the associated codebook.

Coders were partially supervised and required to provide links to validate their coding decisions. Coders evaluated the think tanks and witnesses for false positives as they worked through the list of names. Sixty-three individuals (1.1%) were coded as representing multiple disciplines. For example, Thomas Schelling earned a PhD in Economics, but was a member of Harvard’s school of government and significantly contributed to political game theory; thus earning him recognition as an Economist and as a Political Scientist in Wikipedia. The most common degree among multiple degree holders was Economics, and the most common combination was Economics and Political Science.

Fourth, and finally, we manually reviewed the witnesses from several government organizations known to employ social scientists: the Census Bureau, Congressional Budget Office, Council of Economic Advisers, Federal Deposit Insurance Corporation, Federal Reserve Board, and the National Science Foundation Social and Behavioral Sciences Division. There were 1,687 testimonies from these organizations, and we followed the same protocol described above to identify the disciplinary affiliation of witnesses. These organizations do not exhaust the government organizations that hire social scientists, and searching other organizations would undoubtedly uncover additional missing testimony. However, we believe searching additional government organizations is neither necessary nor practical for capturing broader trends for several reasons. First, we already capture many of the social scientists in other government organizations through our searches of witness disciplines and the names of prominent social scientists. For example, of the 197 social science testimonies from the Census and NSF-SBE, 99 of these testimonies were already in our data, meaning that we added 98 testimonies through manually coding these two organizations, accounting for only around .5% of total testimony in our data. Second, other organizations, such as the National Institute of Justice (NIJ) or National Institute of Health (NIH) only show up five times in our original list of social scientists, suggesting that social science testimony from these organizations is relatively rare. Moreover, the NIJ and NIH hire a great number of lawyers, MDs, public health PhDs, and epidemiologists making them impractical to search through, especially given the limited number of positive cases we see in our original list of names. Even in places like the Census Bureau, economists tend to dominate (economists are the most common (43%) witnesses at the Census Bureau, with sociologists being 34%), and thus adding additional government organizations is unlikely to change the overall picture of disciplinary representation. Finally, as our results below show, even these organizations represent a relatively small share of total representation.

In summary, we identified 15,506 social scientist testimonies from 5,457 individuals, representing roughly 1.8% of overall congressional testimony, using our four major strategies: keyword searches, searching for prominent individuals, manually checking all the testimony associated with think tanks, and searching six government organizations that are staffed heavily by social scientists. In addition to academic and government positions, social scientists testified on behalf of 154 think tanks (1,884 total testimonies). These efforts no doubt missed some testimony from social scientists, but we believe we have identified the overwhelming majority of social scientists who testified before Congress and accurately convey the overarching trends in representation across several sources.

## Social science representation before the United States Congress

**Fig 1:**
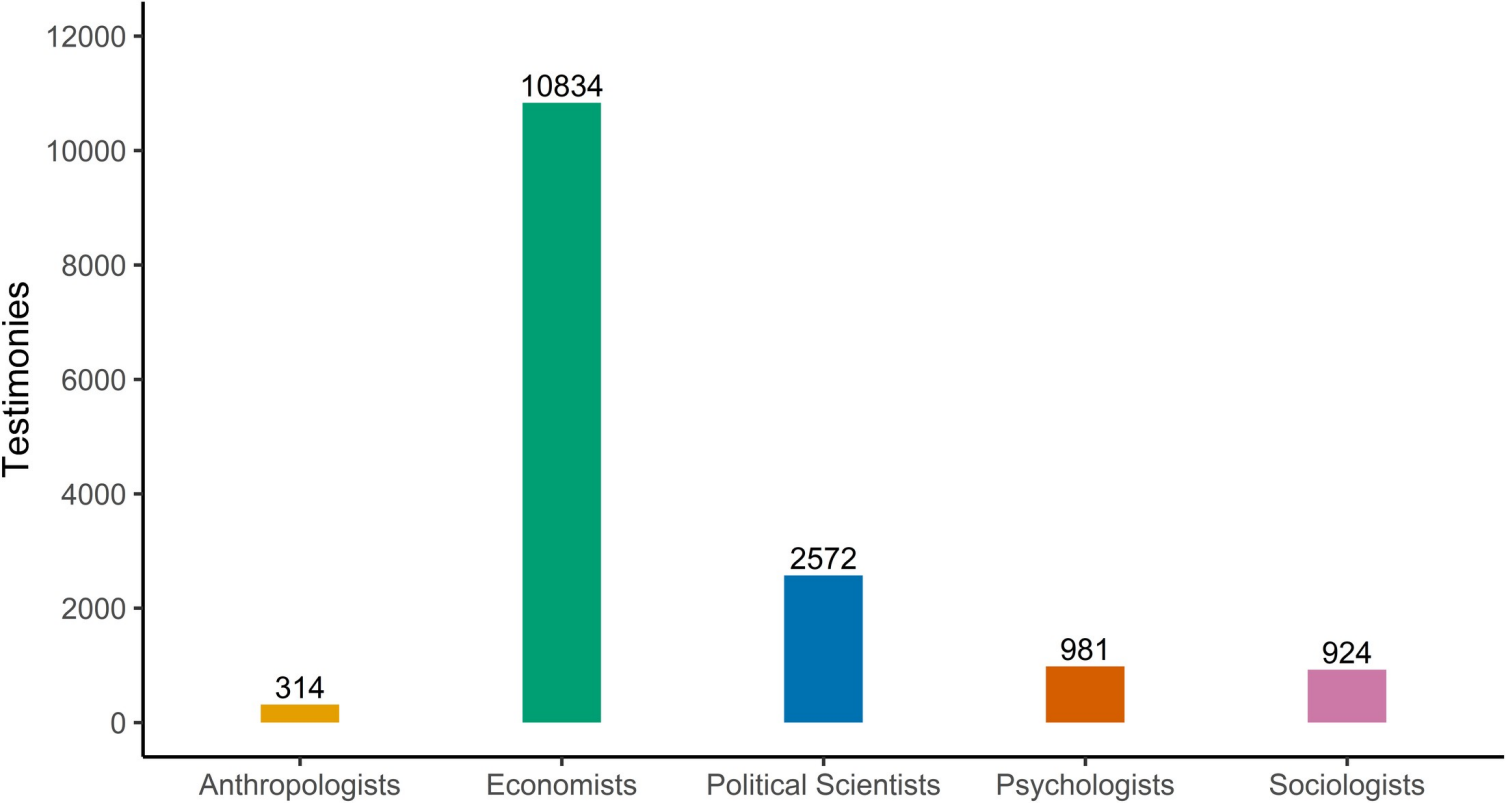
Testimony by discipline at Congress: 1946–2016.

The dominance of economists in social science representation before Congress between 1946 and 2016 is our most striking finding. Economists were more than four times as common as political scientists and more than ten times as common as sociologists (Fig 1). Sociologists’ representation was comparable to psychologists, and significantly more than anthropologists. Yet it paled in comparison to political science or economics.

Although striking, these findings are consistent with previous studies in other domains. Wolfers [7] also finds that economists significantly outpace other disciplines in mentions in the congressional record and New York Times coverage. While he does not include political scientists, the proportional relationship in coverage between historians and economists is comparable. Indeed, if we emulate Wolfers’ search in the Library of Congress search engine [36] while looking for “political science” we find 2309 mentions between 1995 and the present. Despite the discrepancy in references in the Library of Congress [7], sociologists were invited to testify as frequently as psychologists, and more frequently than anthropologists did. These findings are also consistent with previous work arguing for the centrality of economic ideas in policy decision-making processes [4,5,12,37].

**Fig 2:**
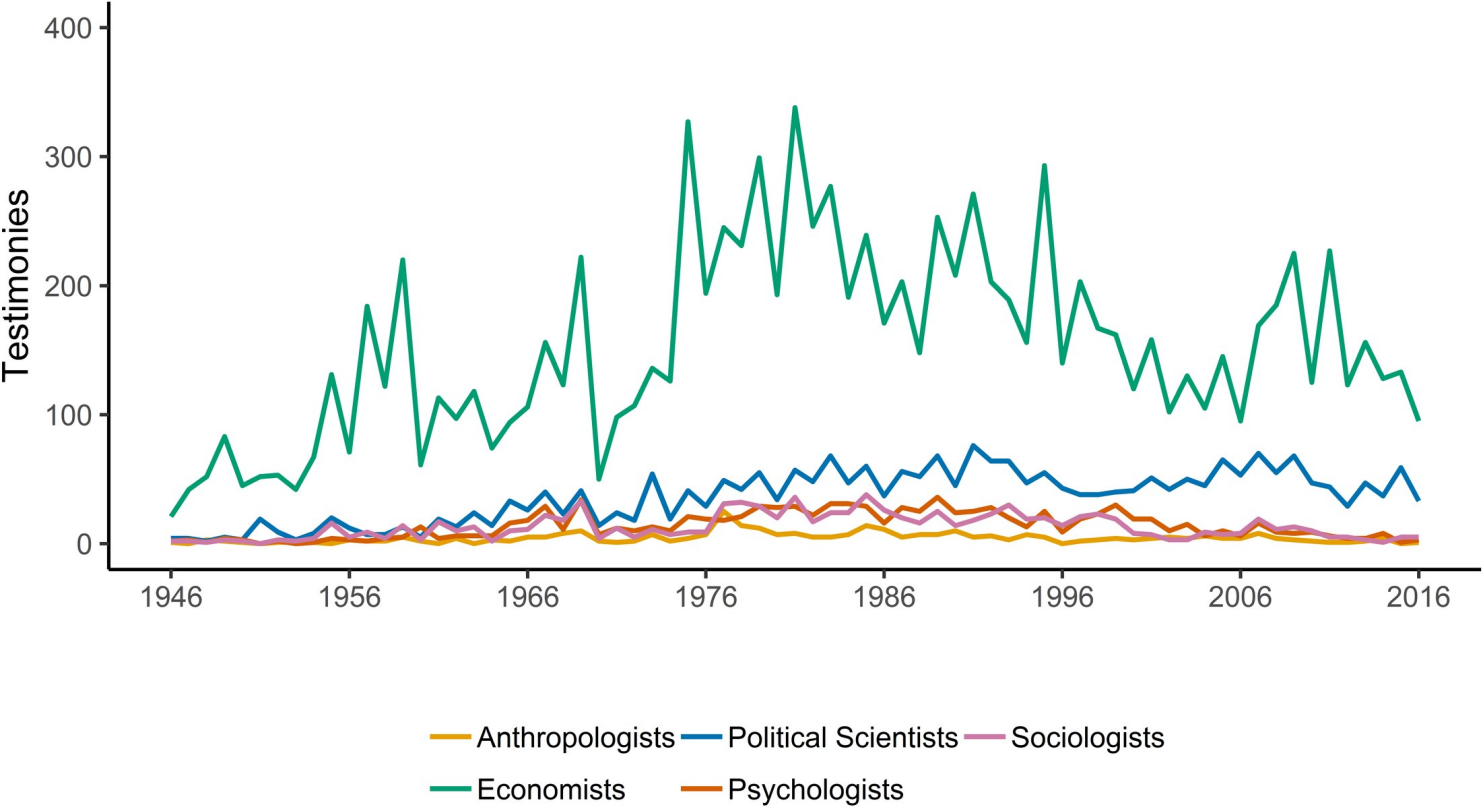
Social scientists testifying before Congress 1946–2016.

**Fig 3:**
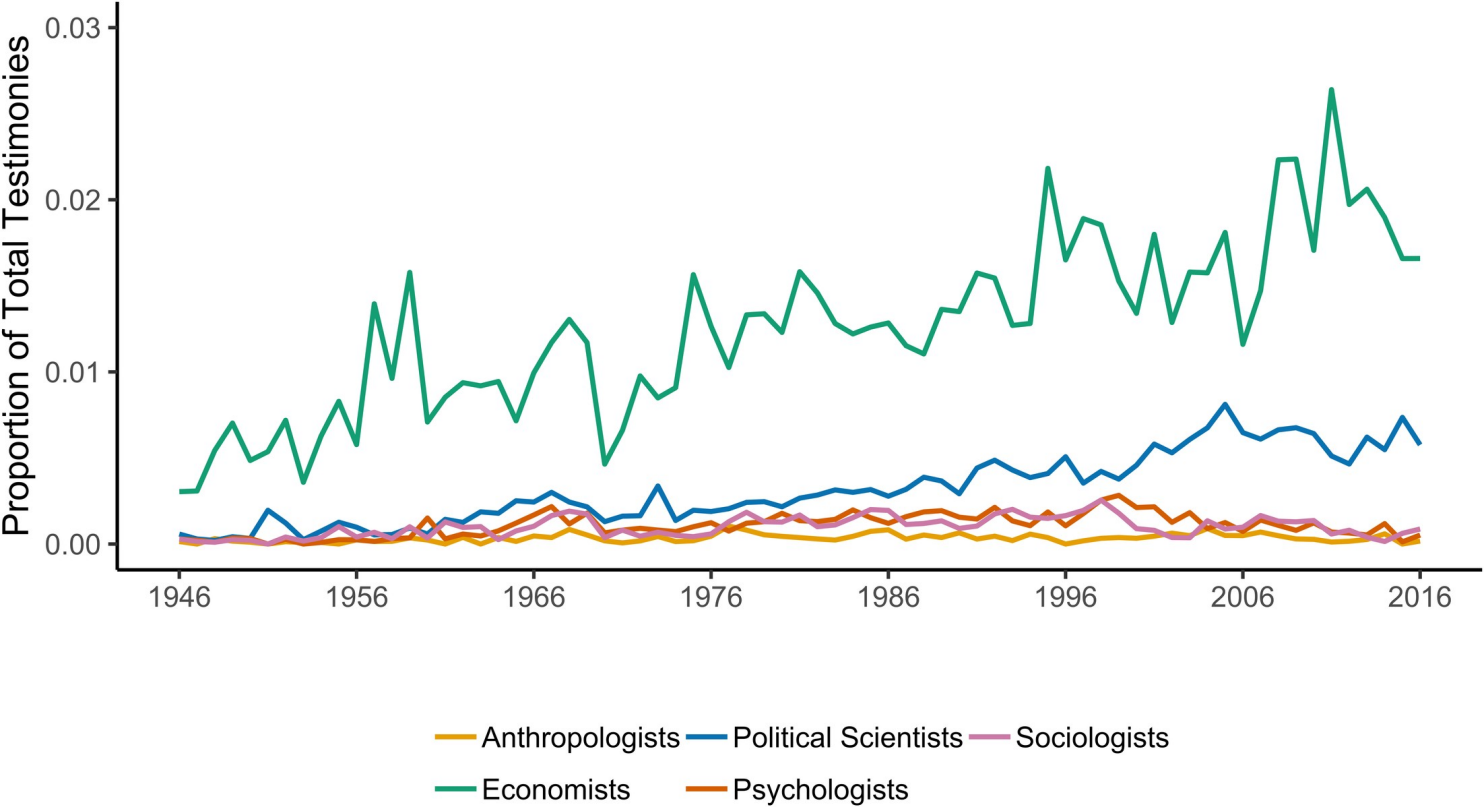
Social scientists as proportion of all testimony.

The dynamics of the total number of witnesses from different social sciences before Congress over time (Fig 2) are consistent with historical accounts. Maasen and Weingart point out that the demands for scientific testimony (and the politicization of science) expanded in the late 1960s [28], and this has not been limited to the hard sciences. Hirschman and Berman point out, for example, that after World War II “economists’ professional authority continued to increase, at least into the 1960s, and economists took advantage of this period to institutionalize their gains” [6]. This period also coincided with the rise of economics in the anti-trust field [2] and its integration into public policy programs [38]. While Fig 2 (and Fig 3 below) shows a decline for other disciplines since the mid-1990s, political science has remained. The centrality of economics and the persistence of political science may be related as we coded public policy as a subset of political science. A portion of the growth seen in Fig 2 can be attributed to the increased representation from scholars with public policy (20% of all political scientists) or international studies (16.5% of all political scientists) training. The growth of public policy (as well as international relations) is consistent with Fleishman’s [38] point regarding the influence of economic thought through its influence on other disciplines [6]. Five hundred and three of the political scientists in our sample were representing think tanks when they testified. As Fig 3 shows, the number of social science testimonies is more stable when normed for the total number of hearings, and political scientists and economists are claiming a steadily increasing share.

Table 1 shows the five most common committees for each discipline. For clarity, we aggregated House, Senate, and Joint committees. We include the specific branch of Congress in the dataset. Each discipline is represented before the Appropriations Committee (economists were invited 442 times, the seventh most common committee). Sociologists were most commonly called before the Judiciary Committee. The majority of political sciences’ invitations came from the Foreign Relations Committee (and the Armed Services Committee) highlighting the fact that their representation before Congress comes mostly from the International Relations (IR) wing of the discipline rather than those focused on domestic politics. Economists were most commonly called before the Economic Committee and the Banking, Housing, and Urban Affairs Committee, but they were invited to testify by non-economic committees—like the Judiciary Committee (611 times) as well.

**Table 1:** Most common committees and counts for each discipline.

Anthropology		Economics		Political Science		Psychology		Sociology	
Name	Count	Name	Count	Name	Count	Name	Count	Name	Count
Appropriations	49	Economic Committee	1617	Foreign Relations	777	Appropriations	159	Judiciary	138
Interior and Insular Affairs	31	Banking, Housing, and Urban Affairs	1148	Judiciary	270	Education and Labor	108	Appropriations	76
Indian Affairs	29	Ways and Means	882	Armed Services	118	Judiciary	83	Foreign Relations	52
Foreign Relations	23	Judiciary	611	Appropriations	101	Labor and Human Resources	56	Post Office and Civil Service	51
Judiciary	14	Finance	603	Economic Committee	65	Labor and Public Welfare	50	Aging	41

### Sources of representation

**Fig 4:**
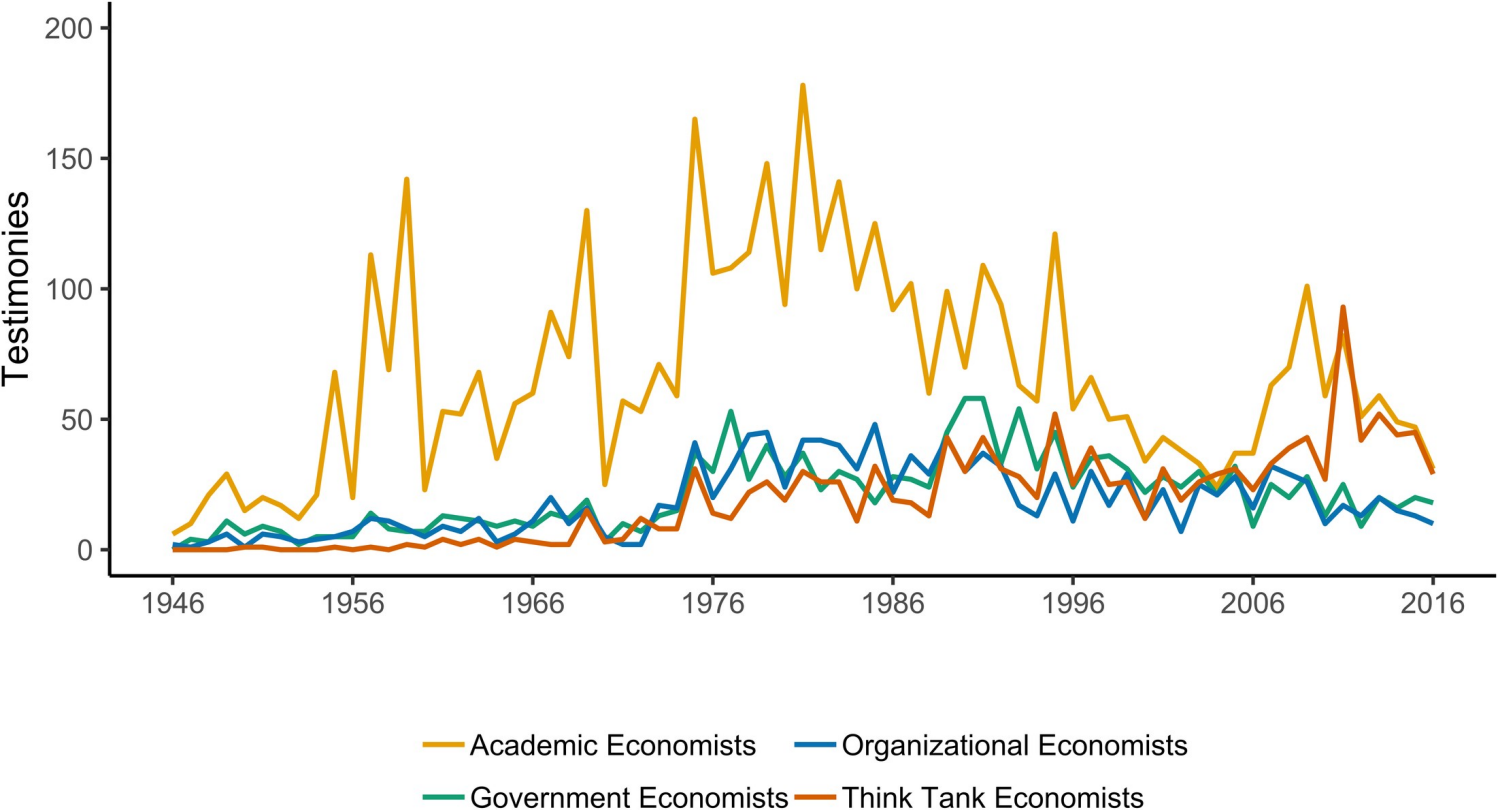
Sources of economist testimony.

We find that social scientists were invited to testify on behalf of a range of institutions, but economists were the dominant discipline regardless of institutional source. Academic economists dominate, but trained economists also staff government positions like the Federal Reserve Board Chair and the Council of Economic Advisers, giving them considerable leverage within the policy field [4,6]. Yet, as Fig 4 below shows, these government agencies hardly exhaust their influence. Economists also testify as “chief economists” or in other capacities for business organizations, labor unions, and other organizations. Increasingly economists also testify on behalf of think tanks.

**Fig 5:**
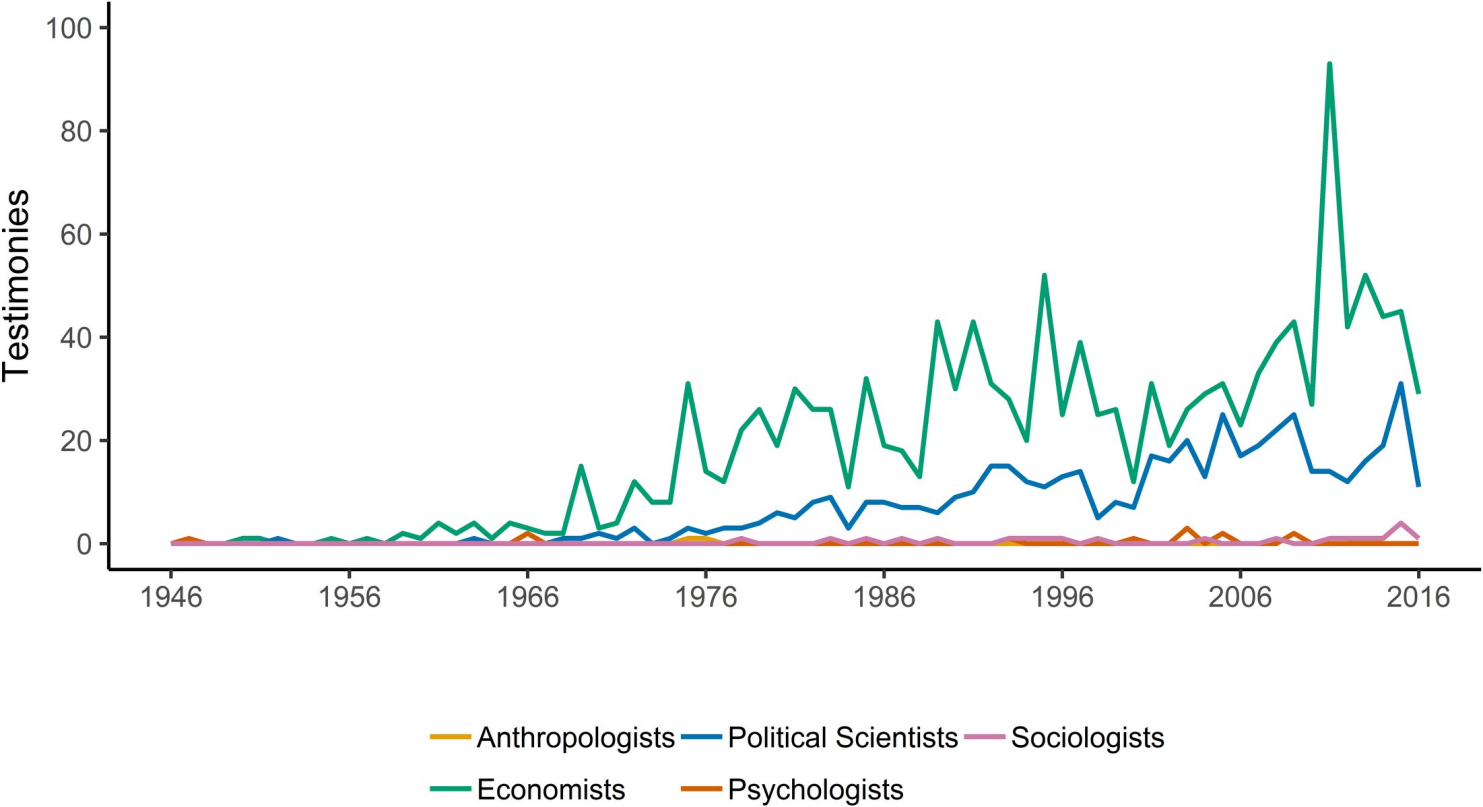
Think tank representation by discipline.

An increasing number of social scientists from other disciplines are also representing think tanks at Congress (Fig 5). Like media coverage [29], congressional attention to think tanks began to rise in the 1960s, but the rise is particularly pronounced in the 21^st^ century as the overall number of congressional witnesses dropped. Think tank witnesses were most likely to have economics training, but political science (often through policy schools) has grown at a steady rate as well.

## Discussion

Although our goal is to provide a broad empirical overview, and introduce new data that can be of use for future explanatory work, we note that our findings do provide some support for existing literature on the complex relationship between politics, science, and expertise. First, our results provide quantitative evidence consistent with the “scientification” of politics, especially after the 1960s [32]. Much of the focus in this area has been on how scientific expertise has expanded as a part of regulatory bodies and research office operations [3,10], and our work shows that this rise is also evident in the congressional deliberation process. As Jasanoff [10] notes, the border between politics and science is often fuzzy within federal regulatory decisions. Our data show the realization of explicit efforts by politicians to integrate social scientists into the political process.

While the scientification of politics has expanded over the past half century, our primary finding—the dominance of economics—is evidence of how policymakers are not drawing evenly from across the disciplines. This provides confirmatory evidence for prior work in sociology that shows how economic logics are more accepted by members of the state [2,6]. Politicians use testimonies to signal their support for ideas or convey information to their colleagues [19,20]. The significant presence of economics is consistent with the claim that their arguments align with how policymakers already think.

Finally, we identify the rise of think tanks as a growing source of expertise for Congress. Think tanks operate at the intersection between politics, academia, business, and markets, and must constantly compete for the attention of politicians in order to sell their ideas and attract funders [30,31]. As a result, they are the organizational embodiment of the “coupling” between science and politics [28,32], and operate in the fuzzy boundaries that Jasanoff identifies [10,31]. Our results show that think tanks are an increasingly important source of social science expertise, and support scholars’ arguments that science and politics are increasingly melded together.

## Conclusion

We presented new, publicly-available, data on the representation of social scientists before the United States Congress, showing that economists give the most testimony to Congress of the social scientists, and they have maintained that advantage even as the sources of testimony have diversified. It is our hope that these data will spur research on the causes and consequences of representation before Congress. Although comparisons are often made to economists, future research might look to how political scientists’ representation before Congress grew while sociology, psychology, and anthropology’s representation remained relatively low. Historical accounts might show how, when, and why congressional testimony by social scientists is, or is not, influential.

We include individual-level data that identifies the specific hearings where individuals appear, allowing for integration of our data with multiple existing datasets. Policy scholars may integrate our data with data on hearing topics and outcomes from the Policy Agendas Project, which may be useful for predicting bill passage, differentiating between policy-making and information gathering within Congress [39], and the influence of the academy (or anti-intellectualism) over time [29].

Future research should look to understand why an increasing share of social scientist’s testimony before Congress is affiliated with think tanks. We caution here that think tanks are not a panacea for increased representation, as Congress and think tanks have a symbiotic relationship that shapes the testimony of think tank representatives: think tanks acquire recognition and Congress uses think tanks to validate foreign, economic, social, and military policy decisions [29,30]. The rise of International Relations think tanks, for example, provides a cautionary tale. IR think tanks generally do not debate grand strategy, but rather provide arguments for American military primacy, which is useful for the politicians and agencies, such as the US Department of Defense, which fund and patronize these think tanks [40,41], meaning that “only the academy can sustain a critique of primacy” [42]. These think tanks also frame alternative grand strategies, as “retreat” or “isolationism,” thus defining alternatives to military primacy as outside the boundaries of legitimate debate [43]. Thus, think tanks may be an effective avenue to get social scientists before Congress, but their testimony may be shaped by the interests of the source organization.

We caution that congressional testimony does not exhaust the pathways to public influence. Scientists have had considerable impacts on other areas of government like the Office of Technology Assessment [3] and regulatory agencies [10], and social scientists may have additional impacts elsewhere in government. Further, Psychologists are likely better represented in medical settings than other social scientists [44]. Sociologists *may* be better represented in community organizations or more local non-profits [45]. At the same time, it is likely that congressional testimony is highly correlated with other measures of representation within the US government, industry, and civil society. Economists, for instance, tend to be better represented in the US news media [7], Silicon Valley [46], and other areas of the US government [6]. Finally, our data does not reflect potential intra-disciplinary effects well. For instance, it may be the case that some of the increasing representation of political science may be as a result of US political science increasingly adopting theory and methodology from economics [47,48]. Despite these limitations, our data afford scholars a unique opportunity to test important theoretical and empirical questions of interest in the future.
